# A proximity biotinylation approach for the identification of membrane contact site proteins in *Toxoplasma gondii*

**DOI:** 10.64898/2026.08.05.743015

**Published:** 2026-08-05

**Authors:** Kaelynn V. Parker, Diego Huet

## Abstract

Inter-organellar communication is crucial for cellular function. Inside the cell, organelles interact with each other via membrane contact sites (MCSs). These structures mediate the close apposition of two organellar membranes to allow for the exchange of metabolites, ions and lipids. Most of what is known about MCSs comes only from a handful of well-studied metazoans, particularly yeast and mammals. Apicomplexans are parasites that drive human disease throughout the world. Yet, little is known about the makeup or function of their MCSs, leaving a gap in our understanding of how organelles communicate beyond conventional model eukaryotes. Here, we used a proximity biotinylation approach to map the surface proteome of three organelles in the model apicomplexan *Toxoplasma gondii* : the apicoplast—a non-photosynthetic plastid found only in apicomplexans—its single mitochondrion and the endoplasmic reticulum. By subtracting a cytosolic spatial reference, our high-stringency proteomic analysis uncovered candidate proteins localized simultaneously to multiple organellar surfaces suggesting their role as MCS components. We then validate our approach by characterizing a candidate involved in the association between the apicoplast and the mitochondrion. Overall, our findings provide a valuable approach to identify MCSs in apicomplexans and set the stage to apply our approach to other organelles in these pathogens.

**Highlights:**

- Generation of surface proteomes for the apicoplast, mitochondrion, and ER in *Toxoplasma gondii*
- Mapping of the first endoplasmic reticulum and mitochondrial surface proteomes in *T. gondii*
- Identified novel membrane contact site candidate proteins
- Validated a membrane contact site candidate mediating mitochondrion–apicoplast interactions

## Full Text Availability

The license terms selected by the author(s) for this preprint version do not permit archiving in PMC. The full text is available from the preprint server.

